# AI-powered mapping of tumor immunity for optimized mRNA vaccine engineering

**DOI:** 10.3389/fonc.2026.1766201

**Published:** 2026-03-03

**Authors:** Ruby Srivastava

**Affiliations:** Department of Chemistry, Indian Institute of Technology (IIT) Bombay, Mumbai, Maharashtra, India

**Keywords:** 5′ UTR, codon optimization, deep learning, immunotherapy, mRNA design, neoantigen prediction, personalized cancer vaccines

## Abstract

Messenger RNA (mRNA) vaccines represent a versatile and scalable platform for cancer immunotherapy; however, their clinical efficacy depends critically on precise vaccine design capable of eliciting robust, selective, and durable antitumor immune responses. Recent advances in bioinformatics and artificial intelligence (AI) have substantially improved the rational design, evaluation, and optimization of mRNA-based cancer vaccines. In particular, personalized vaccine strategies targeting patient-specific tumor neoantigens have demonstrated significant promise, although challenges remain in accurately identifying immunogenic targets within highly heterogeneous tumors and overcoming immune evasion mechanisms. Machine learning and deep learning approaches enhance neoantigen prediction by integrating peptide–major histocompatibility complex (MHC) binding, antigen processing, and T cell receptor recognition, thereby improving immunogenicity assessment beyond conventional pipelines. AI-driven mRNA sequence optimization including codon usage refinement and untranslated region (UTR) engineering further enhances protein expression, translation efficiency, and mRNA stability. In parallel, AI-guided modeling of mRNA secondary structures and lipid nanoparticle (LNP) formulations supports efficient intracellular delivery, improved stability, and controlled immune activation. This review provides a structured overview of AI-enabled computational frameworks for mRNA cancer vaccine development and offers practical guidance for integrating *in silico* predictions with experimental validation. By addressing tumor heterogeneity, antigen processing constraints, and patient-specific immune landscapes, bioinformatics-driven strategies enable more rational and translatable mRNA vaccine design. Collectively, these advances establish a robust foundation for the development of personalized mRNA-based cancer immunotherapies with improved immunogenicity and therapeutic efficacy.

## 1 Introduction

Messenger RNA (mRNA) vaccines have gained prominence as a rapid, adaptable, and scalable platform in cancer immunology, capable of inducing strong and targeted immune responses (1, 2). Despite this promise, translating mRNA vaccine concepts into clinical practice requires overcoming substantial scientific and technical hurdles, emphasizing the need for an interdisciplinary development strategy (1, 3). In oncology, mRNA vaccines embody personalization by targeting patient-specific tumor mutations, marking a major advancement in precision medicine (1). By designing vaccines around individual tumor anomalies, this approach fosters highly specific immune activation with fewer off-target effects, ultimately improving therapeutic outcomes (4). Unlike conventional vaccines that rely on weakened pathogens, mRNA vaccines deliver tumor-associated antigens (TAAs) or neoantigens directly to antigen-presenting cells (APCs), such as dendritic cells or macrophages. Following antigen presentation, adaptive immune responses are activated (5, 6). Neoantigen-derived peptides presented on MHC class I molecules enable the immune system to recognize and target tumor cells (7). Cytotoxic T lymphocytes (CTLs) then eliminate antigen-expressing cancer cells (8), while CD4+ TH1 cells amplify CTL activity and recruit macrophages, shaping a more immunoreactive tumor microenvironment (9, 10). Naked mRNA vaccines can directly enter cells and induce antigen expression but suffer from limited stability and inefficient delivery (11, 12). To address this, lipid nanoparticles (LNPs), currently the only FDA-approved mRNA delivery system encapsulate and protect mRNA, significantly enhancing stability and cellular uptake (13). LNPs facilitate endocytosis, promote cytoplasmic release of mRNA, and boost antigen presentation and CTL activation (14–16). Personalized mRNA vaccine development begins with next-generation sequencing to identify tumor-specific mutations and neoantigens (17, 18). Vaccines are increasingly vital in the face of emerging pathogens and rising antimicrobial resistance (19), yet traditional vaccine development remains slow, costly, and high-risk. The rapid development of mRNA vaccines illustrated how extraordinary resources can accelerate timelines.

Over the past two decades, advances in computational biology have steadily reshaped vaccinology, beginning with reverse vaccinology, which enabled genome-wide antigen discovery at unprecedented scale (20). Recent breakthroughs including machine learning (ML), deep learning (DL), large immunological datasets, and transformative tools have dramatically improved predictive performance and enabled more sophisticated modeling of immune recognition (21). Studies now show that AI models can outperform earlier tools by large margins (22): for example, a B-cell epitope predictor achieving 87.8% accuracy (23) or the MUNIS framework exceeding previous T-cell epitope predictors by 26%; and experimentally validating both known and novel epitopes (24). AI is also accelerating antigen discovery, improving prediction of immunogenicity and safety, and reducing reliance on labor-intensive screening (25). The traditional bioinformatics rapidly identified key antigens such as the spike protein (26) while ML based systems like Vaxign-ML flagged additional targets that had been overlooked in earlier vaccine efforts. DL models trained on hundreds of thousands of human leukocyte antigen (HLA)–peptide interactions now predict T-cell epitopes with accuracy comparable to laboratory assays (23). As AI becomes increasingly integrated into the research and development of vaccine, its practical value for bench scientists lies in accelerating discovery, reducing attrition, and enabling more targeted immunogenicity testing.

In this review, we examine the diverse roles of AI-driven tools in mRNA cancer vaccine design, including applications in sequence generation and data integration, neoantigen identification, codon and UTR optimization, and mRNA formulation and delivery. We also discuss the prevailing challenges, recent advances, and critical future perspectives of mRNA vaccines in precision oncology.

## 2 Sequence generation and data collection

In therapeutic cancer mRNA vaccine development, sequence generation begins with paired tumor–normal genomic profiling rather than pathogen sequencing. Whole-exome sequencing (WES) or whole-genome sequencing (WGS) of tumor and matched normal tissue is used to identify somatic alterations, followed by RNA sequencing (RNA-seq) to confirm transcriptional expression and assess allelic abundance. This dual-layer strategy is essential to distinguish true tumor-specific neoantigens from germline variants and transcriptionally silent mutations. Advanced bioinformatics pipelines integrate variant calling, HLA typing, and transcript quantification to prioritize mutations that are clonally expressed and biologically relevant within heterogeneous tumor populations. Increasingly, spatial transcriptomics and single-cell sequencing are incorporated to resolve intratumoral heterogeneity and to identify immune-excluded or immune-inflamed tumor regions that may influence vaccine responsiveness. Unlike pathogen-centric workflows that emphasize conserved genomic regions, oncology pipelines must explicitly account for tumor evolution, subclonal diversity, and immune escape mechanisms that directly impact neoantigen availability and therapeutic efficacy (17, 27).

Oxford Nanopore enables real-time long-read sequencing, providing access to full-length transcripts and complex genomic regions useful for identifying structural variants (28). PacBio’s high-fidelity long reads enhance detailed viral genome characterization and variant detection. Across these platforms, bioinformatics tools ensure high-quality pre-processing and analysis. FASTQC evaluates sequence quality metrics, while Trimmomatic removes adapters and low-quality regions to improve data reliability (29). Sequence Alignment Map (SAM) tools supports the handling of aligned sequences in SAM/BAM formats, enabling precise variant calling and genomic exploration.

**Figure 1:**
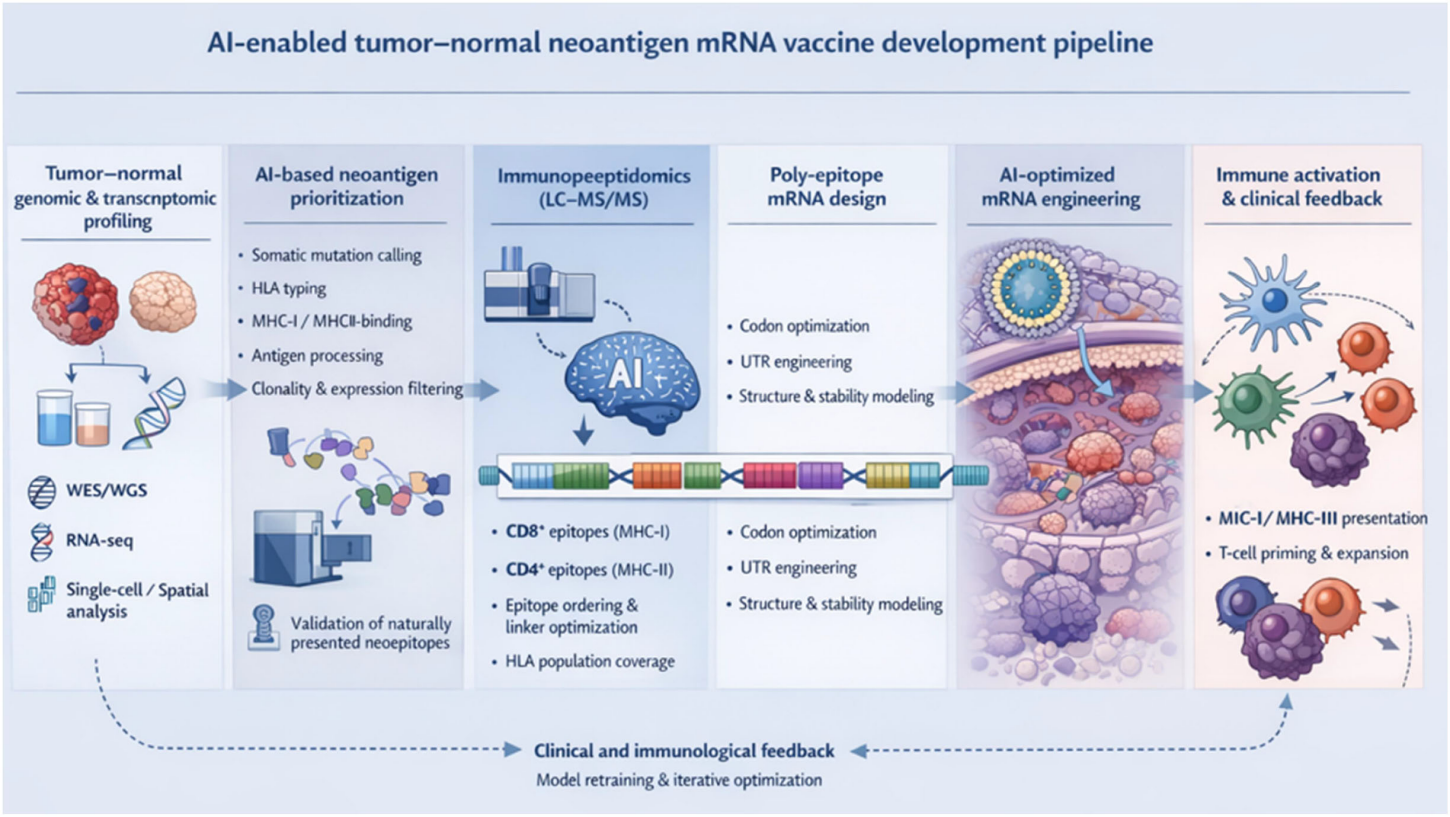
AI enabled tumor–normal neoantigen mRNA vaccine development pipeline.

**Figure 1** schematically illustrates an integrated, end-to-end workflow for personalized cancer neoantigen mRNA vaccine development. The pipeline begins with tumor–normal genomic and transcriptomic profiling using WES/WGS, RNA-seq, and single-cell/spatial analyses to identify tumor-specific somatic mutations and expression patterns.

**Figure 2:**
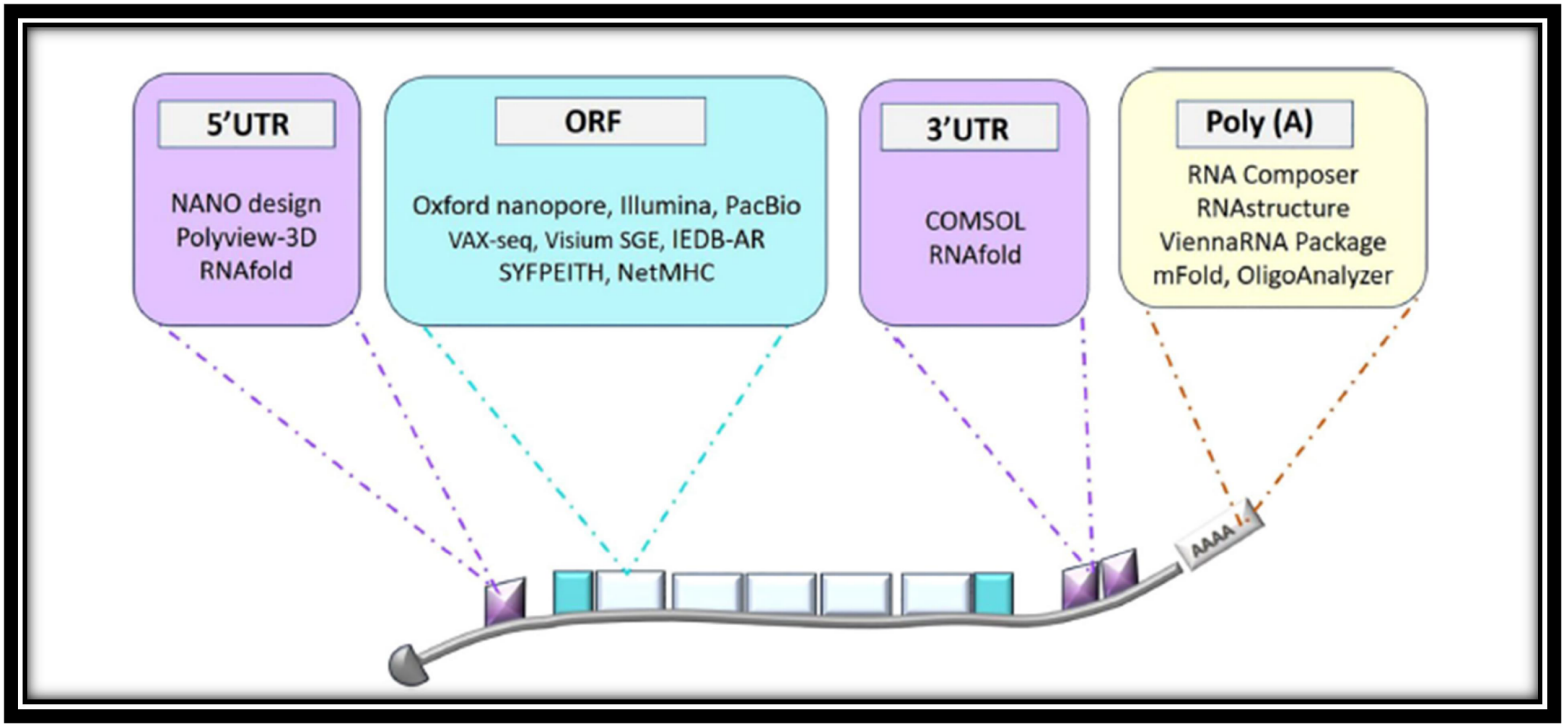
An overview of bioinformatics platforms for mRNA structure prediction and design, illustrating stages such as secondary structure analysis, coding sequence (CDS) optimization, and 3D modeling, along with the tools supporting each step to advance therapeutic mRNA development (32).

Alignment tools such as BWA and Bowtie map mRNA reads to reference genomes (30), and advanced platforms like Visium Spatial Gene Expression (SGE) integrate transcriptomics with histological imaging to reveal spatial patterns of gene expression within tissues (31). These computational approaches help identify conserved regions and immunogenic epitopes. Following alignment, assembly algorithms reconstruct complete mRNA sequences, ensuring the structural integrity of vaccine candidates. In outbreak settings, these workflows can rapidly identify conserved viral elements essential for eliciting protective immunity (32). See **Figure 2**.

Beyond biochemical and immunological factors, the physical and mechanical properties of the tumor microenvironment (TME) play a decisive role in limiting the delivery efficiency of mRNA-loaded nanoparticles in solid tumors. Hallmarks such as increased matrix stiffness, dense extracellular matrix (ECM) deposition, vascular compression, and elevated interstitial fluid pressure (IFP) collectively restrict nanoparticle transport, extravasation, and homogeneous intratumoral distribution. Excessive collagen crosslinking and hyaluronan accumulation increase solid stress and reduce pore size within the ECM, thereby impeding nanoparticle diffusion and limiting access to cancer and immune cells distant from blood vessels (33, 34). Concurrently, vascular compression and abnormal tumor vasculature diminish perfusion and reduce convective transport, while elevated IFP eliminates pressure gradients necessary for nanoparticle penetration, resulting in heterogeneous uptake and reduced therapeutic efficacy (33, 35). These mechanical barriers are particularly detrimental for lipid nanoparticle (LNP)-based mRNA vaccines, whose delivery relies on efficient vascular extravasation, interstitial transport, and cellular internalization. Emerging AI and ML–based models are increasingly being developed to incorporate these physical constraints, integrating imaging-derived biomechanical parameters (e.g., stiffness maps from elastography), histopathological ECM features, and transport simulations to predict nanoparticle penetration and spatial distribution within tumors. Physics-informed neural networks, multiscale modeling frameworks, and graph-based learning approaches have shown promise in linking tumor mechanics with delivery outcomes, enabling the rational optimization of nanoparticle size, surface chemistry, and dosing strategies to overcome transport limitations (36). By incorporating mechanical TME features alongside molecular and immunological data, AI-driven delivery models offer a more realistic representation of solid tumor barriers and support the design of mRNA vaccine formulations capable of achieving improved intratumoral distribution, enhanced cellular uptake, and more consistent immune activation across heterogeneous tumor regions.

Identifying neoantigens capable of provoking a strong immune response remains a complex challenge due to tumor heterogeneity, intricate MHC–peptide–toll cell receptor (TCR) interactions, dynamic tumor evolution, and limited availability of high-quality training data. Conventional neoantigen prediction pipelines incorporate multiple steps including mutation calling, HLA typing, estimation of peptide–MHC–TCR interactions, and immunogenicity prediction often relying on AI, ML, or neural network-based algorithms (37). In contrast, DL approaches offer improved precision by capturing the complexity of tumor genomics and predicting neoantigens across diverse tumor subclones. These models integrate multi-omics datasets such as RNA sequencing, HLA profiles, and whole-exome sequencing to identify neoantigens arising from gene fusions, indels, alternative splicing, and single-nucleotide variants (SNVs). DL architectures, including convolutional neural networks (CNNs) and graph neural networks (GNNs), enhance neoepitope prediction by detecting patterns that traditional algorithm may miss. Additionally, these advanced computational models reduce human error, accelerate analysis, and lower resource requirements, offering a more efficient path toward designing personalized cancer vaccines.

Somatic mutations typically include SNVs, gene fusions (38), insertions and deletions (indels) (39), splice-site alterations (40), intron retention events (41), mutations in non-coding regions (42), exon–exon junction changes (43), and human endogenous retroviral elements. Among these, SNVs are the most widely used source for neoantigen prediction. Several ML tools support this process, including LumosVar (44), DeepVariant (45), GATK 4.0 (46), Cerebro (47), and SpeedSeq (48). LumosVar (44), a Bayesian tumor-only somatic variant caller distinguishes somatic from germline variants by comparing mutation rates, but its performance is strongly influenced by sequencing depth and tumor purity. Although SNVs are commonly used, neoantigens arising from non-SNV alterations may be more immunogenic due to their rapid protein-level changes. Moreover, paired normal–tumor samples are not always available for analysis (45). Wood et al. applied a random forest (RF)-based approach to large datasets, generating high-confidence mutation calls with a predictive accuracy of 98%, supported by automated training on high-quality datasets. However, this approach remains limited by the dependence on NGS datasets (49). Google’s DeepVariant (50) uses deep neural networks (DNN) to classify mutation sites by generating comparative images of normal and tumor reads. It achieves superior performance, with the Food and Drug Administration (FDA) reported F-scores of 99.40% for indels and 99.96% for SNVs compared with benchmarks such as SpeedSeq (48) and GATK 4.0 (46). Although highly accurate, DeepVariant is computationally intensive; its optimized version, DeepVariant-on-Spark (48), reduces processing time through distributed computing. NeuSomatic (51), a DL tool with nine convolutional layers, directly processes raw sequencing data and incorporates feedback from multiple callers. Operating on extensive central processing unit (CPU) and graphics processing unit (GPU) resources, it excels in detecting mutations in samples with low tumor allele fractions but requires longer processing times than DeepVariant. DeepNeo (51) represents one of the most advanced DL models, integrating allelic frequencies, gene expression, and structural features using transfer learning, attention networks, 3D CNNs, and GNNs. It achieves a prediction accuracy of 94%, and clinical trials show a 44% response rate in melanoma patients receiving DeepNeo-based vaccines (51). Despite growing interest in non-SNV-derived neoantigens, especially fusion gene–derived peptides, significant challenges remain in applying AI for their accurate prediction (52).

## 3 AI in neoantigen discovery

While AI-driven antigen discovery gained prominence during the COVID-19 pandemic, its application in oncology presents fundamentally different constraints. Unlike viral antigens, tumor neoantigens arise from stochastic somatic mutations and are shaped by immune selection, antigen processing efficiency, and patient-specific HLA diversity. Consequently, AI models used in cancer vaccine development must integrate mutation clonality, tumor expression levels, peptide processing likelihood, and immune tolerance to accurately prioritize clinically relevant targets (53). DL frameworks trained on tumor-derived immunopeptidomics datasets increasingly outperform conventional motif-based predictors by identifying neoepitopes that are naturally processed and presented on MHC class I and II molecules, a critical requirement for effective CD8^+^ and CD4^+^ T-cell priming in therapeutic vaccination (54). Immunotherapies aim to eliminate cancer cells by activating both innate and adaptive immune responses, and numerous strategies have been extensively evaluated. Because of their strong efficacy and broad clinical applicability, immunotherapies are regarded as some of the most promising approaches for cancer treatment. The antitumor immune response follows several coordinated steps. Initially, cancer cells release tumor antigens that are captured by dendritic cells (DCs), processed, and presented on MHC molecules. These antigen–MHC complexes are then recognized by T cells, leading to their activation and proliferation. Activated effector T cells subsequently identify and destroy tumor cells through TCR–mediated recognition of peptide–MHC complexes, releasing additional tumor antigens and further amplifying immune activation. However, multiple factors can contribute to an insufficient antitumor response in cancer patients. These include: (1) low levels of tumor antigens, limiting effective MHC presentation; (2) impaired recognition by DCs and T cells due to peripheral tolerance; (3) immunosuppressive conditions within the tumor microenvironment; and (4) expression of immune-inhibitory markers on cancer cells that facilitate immune evasion (54, 55). To overcome these barriers and enhance antitumor immunity, diverse immunotherapeutic strategies have been developed. Based on their mechanisms of action, mRNA therapeutics can be classified into four major groups: (1) neoantigen-encoding mRNA, (2) tumor-associated antigen (TAA) mRNA, (3) antibody-encoding mRNA, and (4) immunomodulatory mRNA (53). Identifying clinically relevant neoantigens is the essential first step in designing personalized cancer vaccines (54). Neoantigens arise from non-synonymous mutations or other genetic alterations such as gene fusions that generate novel peptides absent from healthy tissues (55). When displayed on tumor cell surfaces via MHC molecules, these peptides can be recognized as foreign by T cells, initiating targeted immune responses (56). However, only a subset of tumor mutations produce neoantigens capable of binding a patient’s HLA molecules and activating T cells, making accurate prediction of immunogenic candidates a major challenge in vaccine design (57).

Traditional *in silico* workflows typically apply a series of sequential filters: they first identify tumor-specific mutations from exome sequencing data, then predict peptide–MHC binding using tools such as NetMHC (58), followed by filtering candidates based on tumor expression levels and antigen-processing likelihood. Some pipelines also attempt to estimate T cell recognition or overall immunogenicity. Several implementations of these pipelines such as pVACseq (59), MuPeXI (60), and Vaxrank (61) are available, most of which depend on motif-based or ML models for MHC binding prediction while using heuristic criteria for expression and processing steps (59). Although these approaches can generate a list of potential neoantigens, their predictive accuracy remains limited. In real-world settings, only about 1–5% of predicted high-affinity binders correspond to truly immunogenic neoantigens. This low precision results in numerous false positives, increasing the burden of experimental validation and raising the risk of overlooking the most therapeutically relevant targets (59–61).

### 3.1 AI-enhanced neoantigen prediction

AI has been increasingly integrated into neoantigen discovery to enhance performance across every stage of the prediction pipeline. A primary area of improvement has been peptide–MHC binding prediction, given that stable MHC presentation is essential for peptide immunogenicity. Earlier tools such as NetMHCpan (62) already relied on ML, using DNNs trained on large peptide–MHC binding datasets to achieve strong performance for many HLA alleles. More recent advances employ DL architectures capable of capturing complex sequence motifs and, in some cases, structural properties that influence binding. Because MHC alleles differ widely across individuals, they significantly influence peptide–MHC binding affinity. MHC class I and II molecules possess distinct binding groove structures shaped by HLA genotype. MHC class I contains a closed binding groove that accommodates peptides of 8–11 amino acids, enabling stable interaction with TCRs through conserved backbone positions (63). In contrast, MHC class II has an open binding groove that can bind longer peptides (12–20 amino acids), which complicates accurate affinity prediction due to greater sequence variability. Accurate HLA typing is therefore essential before predicting peptide–MHC binding. PCR-Sequence-Based Typing (SBT) remains a standard genotyping method but struggles with polymorphic or heterozygous HLA alleles (64).

Traditional ML methods such as seq2HLA (65), OptiType (66), PolySolver (67), and PHLAT (68) offer cost-effective and accurate HLA genotyping using RNA or DNA sequencing data. OptiType provides highly sensitive predictions for HLA class I using WES or RNA data, whereas seq2HLA and PHLAT predict both class I and II alleles from RNA or WES data. PolySolver, although widely used, is restricted to class I predictions from WES data (67). HLAScan uses DL based CNNs to process raw sequencing reads and predicts HLA class I alleles with up to 99% positive predictive value (69). ATHLATES employs bidirectional long short-term memory networks (LSTMs) to infer both class I and II alleles (70). The most accurate DL framework integrates CNNs and graph neural networks (GNNs) by constructing allele-specific graphs using exons as nodes and linkage disequilibrium as edges; from WGS data. This approach efficiently identifies HLA alleles in population-scale datasets and improves prediction of peptide-binding regions, achieving an 18% success rate in melanoma vaccine design (70).

### 3.2 HLA-based neoantigen prediction

NeoaPred is a DL–based platform that models peptide–HLA class I complex structures *in silico* and evaluates binding interactions and surface-exposed regions to estimate immunogenicity (71). It demonstrates roughly 82% accuracy in reconstructing peptide–MHC complex structures, using these structural features to more effectively identify peptides with immunogenic potential. Such structure-aware methods extend beyond traditional sequence motif–based predictions by incorporating how peptides occupy the MHC binding groove and how much of the peptide is accessible for T cell recognition. Similarly, tools like DeepHLApan (2020) and other CNN and LSTM based models incorporate additional contextual information such as flanking residues or proteasomal processing signals to better predict which peptides are likely to be naturally generated and presented by MHC molecules (72). These DL models, trained on large-scale peptide–MHC interaction datasets (including mass spectrometry (MS) profiles of naturally presented peptides), frequently outperform earlier algorithms in benchmarking studies of binding affinity prediction (73).

Another essential challenge is predicting whether a peptide–MHC complex will be recognized by T cells. Even peptides that bind strongly to MHC may fail to elicit an immune response if they resemble self-peptides or are not recognized by the patient’s TCR repertoire. To address this, several AI models have been developed to evaluate TCR–peptide–MHC interactions. Springer et al. introduced ERGO, an LSTM based recurrent neural network designed to determine whether a specific TCR sequence binds a given peptide–MHC complex (74). ERGO was trained on extensive datasets of known TCR–peptide pairs and outputs a binding probability score. Advancing this further, NetTCR-2.0 incorporated both TCR α and β-chain sequences, employing a CNN architecture to enhance predictive accuracy (75). In this model, peptide sequences and the CDR3 regions of TCRα and TCRβ are one-hot encoded, processed through CNN and pooling layers, and then combined in fully connected layers. NetTCR-2.0 achieved improved specificity and overall performance in identifying true peptide–TCR interactions, surpassing earlier models.

An epitope is the specific part of an antigen recognized by the immune system, with B-cell epitopes targeted by antibodies and T-cell epitopes presented on MHC molecules to T-cell receptors (42). Accurate epitope identification is essential for vaccine design, enabling targeted immune responses. Computational prediction of epitopes accelerates vaccine development and reduces experimental screening, with deep learning models achieving experimental-level accuracy (76). Traditional approaches, including motif or homology-based methods for T-cell epitopes and physicochemical or sequence-conservation methods for B-cell epitopes, often miss novel or conformational epitopes and show limited accuracy (~50–60%) (77, 78). Experimental techniques like peptide microarrays or MS are precise but slow and costly (57, 78). These limitations, including data scarcity and lack of negative controls, make conventional predictions unreliable (79). Modern AI, particularly DL, overcomes these challenges by learning complex sequence and structural patterns from large datasets. Unlike motif-based methods, neural networks capture nonlinear relationships between amino acid features and immunogenicity, improving both B- and T-cell epitope predictions.

Convolutional Neural Networks (CNNs) have been widely applied to predict T-cell epitopes for vaccine design. For example, Deepitope treats all experimentally validated immunogenic peptides as a single class, achieving an Receiver Operating Characteristic Area Under the Curve (ROC AUC) of ~0.59, which improves to ~0.70 when combined with BiLSTM layers (80). In contrast, models like DeepImmuno-CNN explicitly incorporate HLA context, processing peptide–MHC pairs with convolutional layers and physicochemical features, significantly enhancing precision and recall across datasets for cancer neoantigens (81). CNNs also improve B-cell epitope prediction; NetBCE (CNN + BiLSTM with attention) achieved a cross-validation ROC AUC of ~0.85, outperforming traditional tools (82), while DeepLBCEPred (BiLSTM + multi-scale CNN with attention) further improved accuracy and Matthews correlation coefficient (MCC) compared to BepiPred and LBtope (83). These models extract biologically interpretable patterns, highlighting residues critical for epitope recognition. Recurrent neural networks (RNNs), particularly LSTMs, are used to predict peptide–MHC binding and T-cell responses (84). MHC nuggets, an LSTM-based model, predict class I and II peptide–MHC affinity with fourfold higher accuracy than earlier approaches and rapidly evaluates millions of peptide–allele pairs (85). For TCR–epitope specificity, hybrid Attention BiLSTM-CNN models achieve state-of-the-art performance, with AUCs of 0.974 for naïve TCR binding and 0.887 for specific TCR–epitope pairs, surpassing previous models such as TCRGP, ERGO, and NetTCR (86). These DL approaches enable accurate identification of epitopes likely to elicit TCR mediated immune responses.

RNN architectures have significantly advanced linear B-cell epitope prediction. Earlier tools, such as ABCpred, achieved moderate accuracy (~65–67%) (87), whereas more sophisticated models integrate RNNs with additional layers for improved performance. For instance, DeepLBCEPred combines BiLSTM, feedforward attention, and multi-scale CNNs, achieving ~0.67 accuracy and ~0.35 MCC on IEDB benchmarks, slightly outperforming previous methods (83). Ablation studies highlight the BiLSTM as crucial for specificity. Similarly, an attention-augmented LSTM model by Noumi et al. surpassed BepiPred 2.0 by capturing distant sequence dependencies (88). GraphBepi (2023) integrates a BiLSTM sequence encoder with a graph neural network (GNN) operating on 3D antigen structures predicted by AlphaFold2, improving ROC-AUC by >5.5% and some metrics by up to 44% over sequence-only methods (89). BiLSTM or pre-trained language models provide contextual embeddings, while the GNN captures spatial residue–residue interactions, enhancing neoepitope identification.

Graph neural network (GNN) methods have advanced T-cell epitope prediction by modeling peptide–MHC and TCR interactions structurally. GraphMHC (2024) represents MHC–peptide complexes as 3D atomic graphs processed with graph attention and convolution layers, achieving ROC-AUC ~0.92 and outperforming sequence-based methods (90). Its attention mechanism highlights critical interface residues, improving interpretability. Similarly, HeteroTCR (2024) uses heterogeneous graphs of TCR–peptide sequences with multi-hop message passing and attention, significantly enhancing ROC-AUC across multiple datasets (91). These GNN models guide rational antigen selection by linking structural predictions to immunological mechanisms. For B-cell epitopes, structural GNN-based models such as GraphEPN and GraphBepi (with AlphaFold2 structures) improved AUC and Area Under the Precision-Recall Curve (AUPR) by up to 44%, reducing false positives. Collectively, these AI-driven approaches surpass classical methods in accuracy, precision, and discovery of novel, experimentally confirmed epitopes, enhancing vaccine design (86). See **Table 1**.

**Table 1:** Comprehensive overview of AI-driven platforms for B-cell and T-cell epitope prediction, including underlying model architectures, reported performance metrics, and practical accessibility (92).

Tools	Types of Epitopes	AI Architecture	Input	Performance Metrics
MUNIS (2025)	T-cell (HLA-I immunogenic peptides)	Transformer (ESM-2) + BiLSTM hybrid	No	ROC–AUC ≈ 0.980 (median) and AP ≈ 0.289 on test (≈26% better than next best); identified known & novel CD8^+^ epitopes in EBV (exp. validated)
BigMHC (2023)	T-cell (MHC-I binding & neoepitope immunogenicity)	Ensemble of 7 deep NNs(incl. LSTM)	No	ROC–AUC = 0.9733, PR–AUC = 0.8779 for peptide–MHC ligand prediction; after transfer-learning, achieved higher precision than 7 prior models for neoepitope immunogenicity
DeepImmuno (2021)	T-cell (peptide immunogenicity)	CNN (two-stream peptide+HLA)	No	Outperformed earlier tools (IEDB, DeepHLApan) with markedly higher precision and recall on viral and cancer epitope benchmarks (e.g., PR–AUC ≈ 0.62 on an independent set)
BERTMHC (2021)	T-cell (MHC-II peptide binding)	Transformer (BERT fine-tune)	No	ROC–AUC ≈ 0.882 for class II binding (vs 0.877 by NetMHCIIpan); attention heads correctly highlighted key anchor residues
NetMHCpan 4.1 (2020)	T-cell (MHC-I binding affinity)	ANN (pan-specific)	No	Improved class I binding prediction vs prior: e.g., AUROC ~ 0.925 on ligand benchmarks; widely used baseline (covers any HLA-I allele)
NetMHCIIpan 4.0 (2020)	T-cell (MHC-II binding affinity)	ANN (pan-specific)	No	State-of-art for CD4^+^ epitopes until Transformers: e.g., AUROC ~ 0.877 (HLA-II binding); forms baseline for class II prediction
MHCnuggets (2020)	T-cell (MHC-I/II binding affinity)	LSTM network (pan-allelic)	No	AUROC ≈ 0.82, PPV_n ≈ 0.42 on ligand test (≥3× higher PPV than NetMHCpan4.0, MHCflurry, etc.); scanned 26 million peptide–HLA pairs in ~2.3 h (high-throughput)
DeepHLApan (2019)	T-cell (MHC-I neoantigen binding + immunog.)	RNN-based multi-task (binding + immunogenicity)	No	Binding model AUC > 0.90 on 43 HLA alleles (comparable to NetMHCpan); adding immunogenicity filter boosted neoantigen precision by 43.8% (at top 10% rank) (e.g., ~9.6%→13.8% PPV)
MHCflurry (2018)	T-cell (MHC-I binding affinity)	ANN ensemble (allele specific)	No	AUROC ≈ 0.92–0.93 on IEDB benchmarks (comparable to NetMHCpan); top-speed predictor (GPU-optimized) scanning millions peptides/sec
DeepBCE (2023)	B-cell (linear epitope immunogenicity)	CNN (sequence-based)	No	Accuracy = 87.8%, AUC = 0.945, F1 = 0.871 on benchmark (10-fold CV); MCC ~ 0.59 higher than prior state-of-art on average
DeepLBCEPred (2023)	B-cell (linear epitope)	BiLSTM + multi-scale CNN +attention	No	~66–67% accuracy, MCC ~ 0.35 on IEDB tests (slightly above previous ~0.34 MCC); on a larger independent set, outperformed BepiPred2.0 by +0.31 ACC and +0.63 MCC (substantially higher precision)
BepiPred 3.0 (2022)	B-cell (linear & conformational)	Transformer (ESM-2 embeddings + ANN)	No	ROC–AUC ≈ 0.70–0.71 on conformational epitope test (vs ~0.58 by BepiPred2.0/epitope3D); best overall in 2022 benchmark of structurebased tools
EpiDope (2021)	B-cell (linear epitope)	BiLSTM (with dense layers)	No	ROC–AUC ≈ 0.67 (± 0.07) in 5-fold CV, significantly above earliemethods (~0.56 AUC); improved detection of known linear epitopes in tests
GraphEPN (2025)	B-cell (conformational epitope)	VQ-VAE + Graph Transformer (GNN)	Yes	Achieved highest accuracy and MCC on multiple benchmarks (significantly outperforming SEMA2.0, BepiPred3.0, etc.); robust across diverse antigens (uses pretrained structural embeddings)
SEMA 2.0 (2024)	B-cell (conformational epitope)	Ensemble AI (sequence + 3D features)	Yes	Reported ROC–AUC ≈ 0.75–0.80 on structure-based epitope benchmarks (vs ~0.66 by older tools); integrates AI for sequence & surface patch analysis (NAR test winner)
EpiGraph (2024)	B-cell (conformational epitope)	GNN (graph attention) + LM embeddings	Yes	AUC_PR ≈ 0.23–0.24, outperforming BepiPred3.0/DiscoTope on benchmark (vs ~0.19); exploits spatial epitope clustering (graph homophily) to boost precision
DiscoTope 3.0 (2024)	B-cell (conformational epitope)	Inverse-folding transformer + CNN	Yes	AUC–PR ≈ 0.232 on benchmark (vs 0.177 by next-best BepiPred3.0); top F1 and MCC among structure-based predictors in 2024 study
GraphBepi (2023)	B-cell (conformational epitope)	GNN (graph attention) +BiLSTM	Yes	ROC–AUC improved by >5.5% and AUC_PR by ~44% over prior SOTA (e.g., ~0.80 vs 0.75 AUC; PR AUC ~ 0.24 vs 0.17); leverages AlphaFold2 structures for +5–6% absolute gain in accuracy
epitope3D (2022)	B-cell (conformational epitope)	Gradient boosting (graph features)	Yes	ROC–AUC ≈ 0.59 on external test (vs ~0.51 DiscoTope2.0); one of first ML approaches combining structural graph signatures with ML classifier
epitope3D (2022)	B-cell (conformational epitope)	Gradient boosting (graph features)	Yes	ROC–AUC ≈ 0.59 on external test (vs ~0.51 DiscoTope2.0); one of first ML approaches combining structural graph signatures with ML classifier

A major advancement in TCR–peptide–MHC prediction is pMTnet (93), a transfer learning model that first generates embeddings from large general datasets and then fine-tunes on known TCR–pMHC pairs, enabling transfer of knowledge from viral epitopes to tumor-specific neoantigens. Other AI approaches integrate multiple features influencing immunogenicity, such as peptide length, hydrophobicity, and similarity to self-proteins (86). Deep learning pipelines combine MHC binding, antigen processing, and immunogenicity predictions to rank neoantigens, outperforming traditional tools like NetMHCpan and successfully identifying experimentally validated neoantigens in cancer datasets. MHC allele diversity affects peptide binding: MHC class I has a closed groove for 8–11 aa peptides, while class II’s open groove binds longer peptides (12–20 aa), complicating binding predictions (93).

### 3.3 Neoantigen prediction from peptide-MHC I and II binding affinity

Accurate neoantigen identification critically depends on estimating peptide binding affinity to MHC class I and II molecules. Earlier tools relied on matrix-based or linear regression approaches, whereas modern methods employ ML and DL models to capture nonlinear relationships between neoantigen sequences and MHC molecules. For instance, NetMHCpan 4.0 (62) uses allele-specific artificial neural networks trained on ~180,000 mass spectrometry-derived HLA-peptide binding measurements. While effective, it is less generalizable to rare or novel HLA alleles due to biases in training datasets. EpiToolkit (94) is a pipeline that integrates WGS, WES, and RNA-seq data to predict SNV and indel-derived neoantigens. It uses NetChop (95) for proteasomal cleavage prediction and NetMHC (58) for peptide-MHC binding affinity assessment, focusing on MHC class I neoantigens. However, its reliance on pre-established HLA binding models limits prediction of rare neoantigens, and it does not account for post-translational modifications. pVAC-Seq (96) processes WES and RNA-seq data to predict MHC class I neoantigens, combining NetMHC v3.4 (58) for binding affinity, Cufflinks for gene expression, and bam-read count for allele frequency analysis. While comprehensive, it primarily depends on predicted binding affinity and variant allele frequency, and sequencing inconsistencies may affect accuracy (97–99). FRED2 (100) predicts both MHC class I and II neoantigens by integrating multiple tools NetMHC, NetMHCpan and factors such as protease cleavage, mutation coverage, allele frequency, gene expression, and Transporter Associated with Antigen Processing (TAP) transport efficiency. Despite its multi-step design, its predictions are limited by training data quality and incomplete modeling of antigen processing, which can generate false positives (62). CloudNeo (101) evaluates VCF mutation files and BAM-based HLA typing, predicting peptide-MHC class I binding with NetMHCpan. While simple and focused on SNVderived neoantigens, it lacks incorporation of structural and biological peptide properties, limiting prediction accuracy. Tlminer (102) leverages RNA-seq data to predict peptide-MHC I binding. Gene expression is quantified with Kallisto (103), and NetMHCpan predicts binding probabilities. This method, however, does not account for peptide processing, transport, or post-translational modifications, and RNA-seq variability can introduce biases (103). INTEGRATENeo (104) integrates RNA-seq and WGS data, emphasizing gene fusions, and predicts both MHC I and II binding affinities using NetMHC4. This approach enables assessment of peptide–MHC interactions for both classes, providing broader neoantigen coverage (104). AI-driven neoantigen prediction has been streamlined through user-friendly pipelines and web platforms. For example, DeepNeo uses DL on structural and sequence features to predict neoantigen immunogenicity (105). pVACtools integrates multiple prediction methods with expression filtering and optional experimental validation to rank candidate neoantigens. DeepVACPred employs an autoencoder-based deep learning model to design optimal epitope sets for vaccines (106). These tools have improved the success of personalized neoantigen vaccines in clinical trials by increasing prediction accuracy, reducing non-immunogenic peptides, and accelerating vaccine development.

The stability and translation efficiency of mRNA vaccines are tightly linked to their nucleotide sequences, yet codon optimization often involves a trade-off. Leppek et al. proposed combinatorial optimization using models for mRNA secondary structure, in-solution stability, and translation efficiency (107). LinearDesign advances this by optimizing codon usage to enhance protein expression while reducing free energy, using lattice parsing from computational linguistics to identify highly stable and efficient codon arrangements (92). Beyond coding regions, non-coding sequences such as the 5′ UTR are critical for translation regulation. Chu et al. developed a semi-supervised transformer-based model (UTR-LM) trained on endogenous 5′ UTRs, incorporating secondary structure and free energy information. Finetuned for tasks including mean ribosome loading, expression level, and translational efficiency prediction, UTR-LM outperformed benchmarks, generated novel high-performance 5′ UTRs, and identified unannotated internal ribosome entry sites (IRESs), validated via experimental assays (108). AI also supports optimization of mRNA delivery systems. Lipid nanoparticles (mRNA-LNPs) remain a leading platform, with deep learning tools such as AGILE and LiON enabling the design of ionizable lipids and tailored LNPs to enhance delivery to specific cell types. AI further improves microfluidic conditions and lipid ratios, increasing manufacturing efficiency and therapeutic effectiveness. Together, these AI-driven strategies in sequence and delivery optimization are transforming mRNA vaccine development, enhancing stability, translation, and clinical efficacy (108, 109).

## 4 AI in codon optimization for vaccine antigens

After selecting neoantigen peptides, the next critical step especially for DNA or mRNA-based vaccines is encoding them into genetic sequences that can be efficiently translated into proteins. The success of personalized cancer vaccines depends heavily on effective antigen expression and presentation by antigen-presenting cells. Codon optimization is essential to maximize protein expression by altering the coding sequence without changing the amino acid sequence. Software tools such as GeneOptimizer (110) and JCAT (Java Codon Adaptation Tool) (111) facilitate codon optimization by selecting the most efficient codons based on host-specific tRNA abundance and codon usage patterns. GeneOptimizer is a robust platform that optimizes DNA sequences using a sliding-window strategy to refine codon usage, GC content, and other sequence features, ultimately enhancing translation efficiency (112). It is capable of processing large genes and accounts for multiple biological factors, including transcription, splicing, translation, and mRNA stability. JCAT applies advanced metrics such as the Codon Adaptation Index (CAI) and the Relative Codon Adaptation Model to improve heterologous protein expression, without requiring users to manually select highly expressed genes, making the optimization process both efficient and automated. Different organisms exhibit preferences for synonymous codons due to variations in tRNA abundance and regulatory mechanisms. For example, a human gene may not express efficiently in *E. coli* because of codon usage differences. Even within human cells, codon choice can influence translation speed, mRNA stability, and protein folding. Kudla et al. demonstrated that synonymous variants of GFP in *E. coli* displayed up to 250-fold differences in expression, largely due to differences in mRNA secondary structure near the start codon (113). Similarly, the MDR1 gene illustrates how a synonymous C3435T mutation can alter cotranslational folding dynamics, producing a protein with identical amino acids but modified substrate specificity. Studies also show that a “slow ramp” of rare codons at gene starts can enhance translation by preventing ribosomal congestion, while codon autocorrelation can improve efficiency by reusing the same tRNA. Traditional codon optimization approaches rely on heuristics such as the codon adaptation index (CAI), GC content balancing, or removal of problematic motifs like RNA secondary structures or cryptic splice sites (114). However, these methods may miss complex, nonlinear interactions affecting translation. AI-driven codon optimization addresses these limitations by learning optimal codon usage patterns from large biological datasets and predicting the most efficient synonymous substitutions for a given antigen. Significant advancements in AI-driven codon optimization have emerged in recent years. Fu et al. (2020) introduced a BiLSTM-CRF (bidirectional LSTM with a conditional random field) model for selecting optimal codons (112). This approach uses “codon boxes,” which group codons based on nucleotide composition rather than sequence order. By training on highly expressed genes, the model learned preferred codon patterns that enhance protein production. Experimental validation showed that sequences optimized by BiLSTM-CRF achieved substantially higher protein expression than traditional rule-based methods.

Another notable development is CO-BERT (Absci, 2024), a transformer-based language model applied to codon selection (76). Inspired by natural language processing (NLP), CO-BERT treats codons as tokens and learns context-aware representations of coding sequences. By analyzing large genomic datasets, it predicts the optimal codon for a given sequence context, automating what was previously a labor-intensive experimental process and improving protein expression in specific cellular environments. Similarly, CodonBERT (76) was trained on over 10 million mRNA sequences from diverse organisms to capture codon-level preferences. Unlike conventional methods that optimize codons individually, CodonBERT models long-range dependencies to ensure synonymous substitutions do not disrupt regulatory motifs or RNA secondary structures. Tests on vaccine-relevant antigen genes demonstrated that CodonBERT-optimized sequences produced higher protein yields compared to industry-standard approaches. Generative models have also been applied to codon optimization. RiboCode is a deep generative framework that incorporates ribosome profiling data to design codon sequences enhancing ribosome loading and elongation efficiency (115). Using reinforcement learning, RiboCode iteratively refines codon sequences to maximize translation efficiency while minimizing structural constraints. Initial results indicate that RiboCode generated sequences outperform heuristic-based methods, yielding higher protein expression. The effectiveness of AI-driven codon optimization has been confirmed in several experimental studies. AI-guided codon optimization can ensure that mRNA constructs encoding patient-specific neoantigens generate high antigen levels in dendritic cells, promoting stronger immune responses. This is particularly important for multivalent mRNA vaccines, where balanced expression of multiple antigens is critical. By optimizing codon usage, AI models help prevent translation competition among different antigenic segments, ensuring that each neoantigen is efficiently produced.

## 5 AI in UTR sequence generation and mRNA design

The stability and translational efficiency of mRNA are key determinants of the effectiveness of mRNA-based cancer vaccines. While codon optimization improves coding sequence efficiency, regulatory elements such as untranslated regions (UTRs) play a major role in overall antigen expression. The 5′ UTR is critical for translation initiation, influencing ribosome scanning and start codon recognition (116), whereas the 3′ UTR modulates mRNA stability, localization, and translation efficiency through interactions with RNA-binding proteins and microRNAs. Traditionally, UTR optimization relied on empirically selecting sequences from highly expressed genes, but AI-driven methods now enable the *de novo* design of synthetic UTRs with enhanced stability and translational performance.

Designing poly-epitope mRNA constructs for cancer vaccination requires careful consideration of epitope ordering, linker composition, and HLA coverage. Unlike viral vaccines that target conserved antigens, cancer vaccines must accommodate extensive patient-specific HLA polymorphism and tumor heterogeneity. AI-guided optimization strategies increasingly incorporate population-level HLA frequency data, predicted peptide–MHC stability, and immunodominance modeling to design epitope cassettes that maximize CD8^+^ and CD4^+^ T-cell responses without competitive suppression. Computational simulations further assist in balancing MHC class I and class II epitopes to promote helper T-cell support, which is essential for sustaining cytotoxic responses and immunological memory in solid tumors.

### 5.1 AI-driven 5′ UTR optimization

In the context of cancer immunotherapy, optimization of mRNA untranslated regions (UTRs) is evaluated not by antibody titers but by their impact on antigen processing, MHC presentation, and T-cell priming efficiency. Sustained yet controlled antigen expression is particularly critical for effective cross-presentation by dendritic cells, which governs the magnitude and durability of CD8^+^ cytotoxic T-cell responses. AI designed 5′ and 3′ UTRs can modulate translation kinetics and mRNA stability to favor proteasomal processing and optimal peptide loading onto MHC class I and II molecules. These features are especially important for multivalent neoantigen vaccines, where balanced expression of multiple epitopes is required to prevent immunodominance and ensure broad T-cell repertoire engagement across diverse HLA backgrounds (116, 117).

The 5′ UTR plays a critical role in translation initiation by affecting ribosome scanning efficiency, start codon recognition, nucleotide composition, secondary structure, and upstream open reading frames (uORFs). AI-driven models have been developed to design 5′ UTRs that maximize translation efficiency while minimizing inhibitory structural elements. GENCODE v19 annotations (118) show that only about 30% of human 5′ UTRs are 100 nucleotides or shorter, with an average length of roughly 200 nucleotides (118). This extensive sequence configuration space (e.g., ~4^200^) makes efficient optimization or design of 5′ UTRs for mRNA vaccines highly challenging. To address this, a growing body of ML methods has been developed to model, optimize, and design 5′ UTRs that influence protein expression. These approaches span a wide range of techniques, including random forests (RF), CNNs, generative adversarial networks (GANs), and more recently, RNA language models (LMs) (119–122). Recent advances in machine learning have enabled systematic modeling and engineering of 5′ UTRs to control translation efficiency (TE). Optimus 5-Prime, a CNN trained on MPRA-derived polysome profiling data from 280,000 synthetic sequences, explained up to 93% of mean ribosome load (MRL) variation but was limited to fixed-length inputs (120). FramePool addressed this by using frame-dependent pooling to generalize across variable-length UTRs, achieving markedly higher performance on diverse-length test sets (r = 0.901 vs. 0.743), though correlations with endogenous TE remained low due to CDS and 3′ UTR influences (121). Additional strategies include random forest modeling of natural UTR features to design highly expressive synthetic UTRs, and multi-task learning (MTtrans), which integrates multiple datasets through shared CNN encoders, improving generalization and identifying conserved regulatory motifs (122). Generative approaches such as UTRGAN produce realistic UTRs optimized for high MRL/TE using adversarial training and gradient-based latent space optimization. More recently, RNA language models such as RNA-FM (123) and UTR-LM (108), pre-trained on millions of sequences, have outperformed previous CNN-based models, with UTR-LM achieving the strongest MRL and TE predictions and experimentally validated design of high-efficiency UTRs that increased protein expression by up to 32.5%, highlighting the power of large-scale pre-training for 5′ UTR functional modeling and design.

A leading example is Smart5UTR, a deep generative model introduced by Tang et al. (124), which design synthetic 5′ UTRs optimized for mRNA vaccine translation. Smart5UTR was trained on a massively parallel reporter assay (MPRA) dataset of over 200,000 randomized 5′ UTR sequences, each experimentally tested for translation efficiency. Using a multitask autoencoder with a CNN encoder, the model learned hidden sequence features that enhance translation initiation. When applied to SARS-CoV-2 spike mRNA vaccines, Smart5UTR designed UTRs significantly increased antigen expression in mice, producing up to 120-fold higher antibody titers against viral variants compared to conventional UTRs, while maintaining a favorable safety profile. Similarly, Castillo-Hair et al. (125) optimized 5′ UTRs for mRNA-encoded gene-editing enzymes. Using polysome profiling of large randomized 5′ UTR libraries, they measured translation efficiency across different cell types and trained a DL regression model to predict UTR sequences that maximize ribosome loading. AI-designed UTRs improved the activity of megaTAL nucleases in human cells, demonstrating enhanced functional protein output. Another approach, UTR-LM, developed by Chu et al. (126), is a pretrained transformer language model that learns sequence representations of endogenous 5′ UTRs from multiple species. Fine-tuned to predict translation initiation efficiency (TIE), UTR-LM identified novel synthetic UTRs that outperformed natural counterparts in driving protein expression. Its cross-species generalization suggests broad applicability for optimizing vaccine mRNAs in diverse expression systems. These AI driven methods illustrate the power of computational design in improving translation efficiency, enabling the development of highly expressive and effective mRNA vaccines.

### 5.2 AI-driven 3′ UTR optimization

The 3′ UTR plays a key role in regulating mRNA stability, degradation, and localization. While conventional vaccines often utilize 3′ UTRs from highly stable endogenous transcripts, such as α-globin, AI-driven approaches enable the design of synthetic 3′ UTRs with enhanced stability and translational efficiency. Morrow et al. (127) developed a machine learning model trained on high-throughput RNA stability assays, using thousands of synthetic 3′ UTR sequences measured for mRNA half-life in human cells. The model predicted stability based on sequence features and generated *de novo* 3′ UTR designs that extended mRNA half-life beyond standard endogenous UTRs. Experimentally, these AI-optimized 3′ UTRs produced prolonged antigen expression in mammalian cells, an important factor for cancer vaccines requiring sustained immune stimulation. Similarly, Liu et al. (128) introduced LinearDesign2, an AI framework that simultaneously co-optimizes the 5′ UTR and coding sequence to improve both translation initiation and mRNA stability. Unlike traditional approaches that optimize UTRs and coding regions separately, LinearDesign2 employs a multi-objective optimization strategy, balancing translation efficiency with mRNA folding stability. Computational analyses indicated that joint optimization of the 5′ UTR and coding sequence yields higher protein expression than optimizing either component alone.

### 5.3 Implications for mRNA vaccine design

AI-driven UTR and mRNA design significantly enhances personalized cancer vaccines by boosting antigen expression, which can double or triple vaccine potency without increasing mRNA dose, improving T cell activation. It overcomes biological constraints, such as problematic RNA structures or cryptic motifs, by designing custom 5′ and 3′ UTRs for robust antigen production. Additionally, AI enables context-specific optimization, tailoring UTRs to different cell types like dendritic cells, and allows integration with codon optimization to simultaneously refine coding regions, UTRs, and RNA structures, advancing mRNA vaccine efficacy (129). Unlike DNA, RNA is highly susceptible to base-catalyzed hydrolysis due to the 2′-hydroxyl group on its ribose, leading to spontaneous degradation especially in exposed single-stranded regions (130). As a result, mRNA vaccines require ultra-cold storage to preserve stability, although sequences with more structured regions, fewer single-stranded motifs, and higher GC content degrade more slowly and may permit more flexible storage conditions (37, 131). Because codon choice can dramatically alter ORF folding stability, ORF design prioritizes sequences that form stable low-MFE structures to enhance mRNA durability (100). Traditional tools such as CDSfold use dynamic programming to identify ORFs with optimal 2D structures (132), but their O(L³) time and O(L²) space complexity make them slow for long mRNAs. LinearDesign overcomes this bottleneck by applying a lattice-parsing strategy adapted from computational linguistics, enabling rapid left-to-right dynamic programming and reducing runtimes from hours to minutes.

Accurate RNA structure prediction remains essential for evaluating molecular stability, and most current approaches focus on two-dimensional structures using thermodynamic models such as the nearest-neighbor framework and Turner parameters (133), as implemented in widely used tools including RNAfold within the Vienna RNA package (134) and Mfold (135). ML provides an alternative route for estimating RNA free-energy parameters (136). Nearest-neighbor ML models such as ContextFold (137) and CONTRAfold (138) learn thermodynamic parameters directly from large RNA sequence–structure datasets. ContextFold can train up to 200,000 fine-grained energy parameters, whereas CONTRAfold uses conditional log-linear models to learn approximately 300 parameters. Although these approaches improve parameter estimation, they are prone to overfitting and often generalize poorly to unseen RNAs (138). Probabilistic generative methods such as Pfold, which relies on stochastic context-free grammars (SCFGs) (139), and TORNADO, which integrates SCFGs with Turner energy parameters (140) offer alternative frameworks but remain limited by high computational complexity and difficulty in modeling intricate RNA features (140). Hybrid thermodynamic ML approaches including SimFold (141), MXfold (142), and MXfold2 (143) combine experimentally derived free-energy parameters with DL based corrections. By training on RNA triplets, free energies, and known structures, these models achieve higher accuracy with reduced risk of overfitting. DL has also enabled new secondary structure predictors that operate via binary classification rather than explicit thermodynamic modeling. Tools such as IPknot (144), E2Efold (145), UFold (146), and SPOT-RNA (147) uses architectures including integer programming, convolutional networks, and triplet-based feature extraction to predict base-pairing patterns. Despite strong performance, these models typically require large training datasets and depend heavily on dataset partitioning strategies (148). Among current secondary-structure predictors, MXfold2 (143) stands out for combining DL with nearest-neighbor thermodynamics and the Zuker algorithm, resulting in robust predictions of stable secondary structures using Turner parameters. For tertiary structure, the recent DeepFoldRNA model (149) represents a major advance, predicting atomic-level 3D RNA structures directly from sequence. Using self-attention neural networks, geometric-restraint prediction, and L-BFGS–based structure optimization, DeepFoldRNA infers inter-residue distances, torsion angles, and folding constraints without relying on homologous templates. Evaluated on 105 complex RNA structures (70–250 nt) from 32 Rfam families and 17 RNA-Puzzles targets, DeepFoldRNA achieved the best performance on 15 of the 17 puzzles, with an average RMSD of 2.69 Å and a TM-score of 0.743 substantially outperforming previous models. A more advanced version, DeepFoldRNA 2.0, is currently under development to further improve accuracy (150).However, these models still struggle with complex features like multibranched loops and pseudoknots due to incomplete thermodynamic parameterization, making consensus predictions across multiple tools a practical strategy. Beyond traditional folding energy, average unpaired probability (AUP) provides an alternative measure of mRNA structural stability, reflecting overall “unstructuredness” and correlating with hydrolytic degradation (150). Using AUP as the objective, Wayment-Steele et al. developed RiboTree, which applies Monte Carlo tree search and LinearPartition to identify ORFs with significantly improved in-solution stability, predicting more than a twofold increase in mRNA stability (151). Recent ML models have improved prediction of degradation and local structural features for short ORFs (152–156), but they still fall short of traditional RNA folding algorithms, particularly for artificially designed or long mRNAs, as highlighted in CASP15 (157, 158).

Local RNA structures such as loops, hairpins, and pseudoknots strongly influence in-cell mRNA stability, translation rate, and fidelity (159). Cellular RNases target specific sequence and structural motifs, with single-stranded regions being particularly vulnerable (151), aligning with the goal of minimizing AUP to extend in-cell lifetime. However, global stabilization alone is insufficient: highly stable structures or rare codons can slow ribosome movement, trigger ribosome collisions, and activate RNA decay pathways, shortening mRNA lifetime and reducing protein yield (160–164). Translation elongation also affects protein cotranslational folding, and excessive codon optimization can disrupt native folding kinetics, producing misfolded or even toxic proteins (165–168). Current ORF design tools do not explicitly model such local structures, suggesting that integrating consensus predictions from multiple physics and ML based RNA structure predictors may help identify problematic regions.

Protein structure prediction is fundamental to mRNA vaccine design because it helps ensure that the antigens encoded by the mRNA fold correctly and function as intended in the host. Unlike expensive and indirect proteomics approaches such as GC-MS, computational prediction provides rapid, theoretical insights that refine antigen design before experimental validation. Tools like AlphaFold (21) and Rosetta are central to this process (169). AlphaFold uses DL particularly attention mechanisms and evolutionary coupling to predict high-resolution 3D protein structures directly from amino acid sequences (21). In mRNA vaccine development, these predictions guide the design of stable, immunogenic antigens and help assess viral antigenicity (170). AlphaFold also informs optimization of mRNA–LNP formulations by predicting structural motifs, such as stem–loops, that improve mRNA stability, nuclease resistance, and release kinetics within target cells. These improvements support enhanced dendritic-cell activation and stronger adaptive immune responses. 176) Rosetta, another leading platform, relies on energy-based modeling, including Monte Carlo sampling and the Rosetta Energy Function, to predict and refine protein structures (169, 171). It can analyze protein interactions, model antibodies and antigens, and interpret glycans from structural data, making it valuable for designing antigen-coding mRNA sequences that elicit robust immune responses. Although highly versatile supporting predictions for proteins from 10 to ~1000 residues (172–175), Rosetta requires careful parameterization, and its accuracy may decline for very large or complex structures.

Molecular dynamics (MD) simulations are essential for understanding the complex movements and interactions of atoms within mRNA molecules, particularly their interactions with proteins and other cellular components. These simulations rely on several critical parameters: force fields define atomic interactions, while temperature and pressure controls replicate physiological conditions (176). Lipid membrane permeability is incorporated to accurately model molecular interactions (177). Time steps balance simulation accuracy with computational efficiency, cut-off distances manage non-bonded interactions, and periodic boundary conditions minimize edge effects to enhance model realism. Solvent models replicate the surrounding aqueous environment, while electrostatic treatments capture long-range interactions, ensuring comprehensive modeling of mRNA behavior. Extended simulation times and advanced techniques, such as replica exchange (178), provide deeper insights into mRNA dynamics. Optimizing these parameters is critical for ensuring that mRNA vaccines retain their structure and function, ultimately enhancing immunogenicity and efficacy. Several MD simulation tools are widely employed in mRNA vaccine development. GROMACS is a leading platform known for its computational efficiency and precision. It supports MD, stochastic dynamics, and path integration methods, enabling simulations of molecules in solution or crystal, energy minimization, and conformational analysis. GROMACS includes diverse force fields suitable for proteins, nucleotides, sugars, and other biomolecules, modeling atomic and molecular movements across systems ranging from glasses and liquids to polymers and biomolecular solutions (179). AMBER (Assisted Model Building with Energy Refinement) is widely used for predicting and refining 3D mRNA structures. AMBER excels in performing detailed energy calculations and structural analyses, leveraging parallel computing and advanced algorithms. Integration of the Generalized Born model and Particle-Mesh Ewald (PME) method ensures accurate treatment of molecular interactions. Its variety of force fields, including specialized options such as ff14SB and nucleic acid-specific fields, allows precise modeling of proteins and nucleic acids, providing critical insights into mRNA vaccine behavior (180). NAMD leverages advanced parallel computing to perform large-scale simulations, capable of modeling millions of atoms. This is particularly valuable for neo-antigen mRNA vaccine design, as it enables precise simulation of interactions between mRNA and proteins, critical for predicting antigen presentation and immune responses (181). NAMD employs sophisticated force fields such as CHARMM and the PME method to calculate long-range electrostatic interactions accurately and efficiently. Its Multiple Time-Step (MTS) integrator allows the simulation of dynamic behavior across multiple time scales, providing insights into the temporal evolution of mRNA-protein interactions (182, 183). Rosetta is a versatile molecular modeling platform primarily used for protein structure prediction, protein-protein and protein-ligand interactions, and biomolecule design. It can also model RNA molecules in 3D. Unlike traditional MD tools, Rosetta uses energy functions and Monte Carlo sampling to explore molecular interactions, predicting large-scale conformational changes with all-atom force fields (173). Its unique capabilities make it valuable for integrating mRNA sequences with protein components to optimize vaccine design. CHARMM (Chemistry at HARvard Macromolecular Mechanics) is a comprehensive MD simulation package for detailed analysis of biomolecular systems. Using advanced force fields such as CHARMM36 and the CHARMM General Force Field, it accurately models nucleic acids and protein interactions, facilitating the study of mRNA stability and behavior within vaccine constructs (184).

## 6 AI in mRNA vaccine formulation and delivery

Optimizing mRNA vaccine sequences is critical, but efficient delivery and stability *in vivo* are equally essential. mRNA vaccines must be translated efficiently into proteins while avoiding premature degradation by extracellular RNases or overactivation of the immune system. LNPs are the primary delivery vehicles, protecting mRNA and facilitating cellular uptake (185), while immune-stimulatory adjuvants further enhance vaccine efficacy (186). AI is increasingly employed to optimize both LNP formulations and adjuvant design to maximize performance. LNPs typically comprise ionizable lipids, phospholipids, cholesterol, and polyethylene glycol (PEG) lipids, with their composition and ratios affecting encapsulation efficiency, stability, and cellular uptake (187). In the context of mRNA-LNP delivery, IPKnot’s folding predictions are essential for optimizing the interaction between mRNA and LNPs. The predicted mRNA secondary structure influences the mRNA’s ability to be encapsulated into LNPs, as well as the subsequent release and translation inside the target cell. IPKnot aids in designing mRNA sequences with secondary structures that are compatible with LNP formulations, enhancing encapsulation efficiency and promoting stable, controlled release into the cytoplasm (188). This stability is vital for maintaining the functional integrity of mRNA once inside the cell, ensuring that it can be efficiently translated to produce the encoded protein.

Traditionally developed by trial and error, AI now accelerates LNP optimization. Mekki-Berrada et al. (2021) applied a two-step machine learning strategy, combining unsupervised clustering of combinatorial nanoparticle data with supervised regression to predict mRNA delivery efficiency, enabling rapid identification of highly effective formulations (189). Similarly, AGILE (2024) uses a GNN trained on thousands of LNP compositions to predict transfection potency, outperforming conventional heuristic approaches (188). TransLNP, a transformer-based model, integrates coarse-grained sequence and fine-grained spatial features to identify optimal LNP structures and transfection cliffs, addressing data scarcity and variability (153). LNPs, ranging from 70–200 nm, are the only FDA-approved carriers for mRNA vaccines, essential for encapsulating, stabilizing, and delivering mRNA into target cells. Their composition, lipids, cholesterol, and PEG directly affects efficiency, with lipid headgroup interactions and hydrophobic tail arrangements critical for membrane fusion and payload delivery (154).

Designing LNPs for mRNA cancer vaccines requires advanced computational tools. NANOdesign enables optimization of lipid types, ratios, particle size, surface charge, and hydrophilicity to maximize mRNA encapsulation, stability, and controlled release (155). It also simulates formulation effects on nanoparticle uniformity and delivery efficiency. POLYVIEW-3D allows 3D visualization of LNPs and their interactions with mRNA and cellular membranes (156). It aids in analyzing nanoparticle morphology, membrane uptake, and interactions with immune cells like dendritic cells, as well as modeling nanocomposite hydrogels to enhance stability and delivery (190). PyProtif (191) uses molecular lipophilicity potential to model interactions between lipids and mRNA, helping optimize lipid composition, surface properties, and particle morphology for efficient encapsulation, cellular uptake, and release. It can also simulate environmental effects, such as pH and temperature, predicting LNP behavior during storage, transport, and administration (192, 193). Together, these tools enable rational design of LNPs, improving the stability, efficacy, and delivery of mRNA vaccines, as demonstrated in recent studies on mRNA vaccine formulations (92).

Beyond delivery, AI is advancing adjuvant design to enhance immune responses. Adjuvants, including TLR agonists, cytokines, or nanoparticle-based formulations, stimulate immune activation. Chaudhury et al. demonstrated that machine learning models, integrating serological, cellular, and cytokine data, could predict adjuvant conditions with up to 92% accuracy (194). Using AI-guided design, a library of AuNP-based adjuvants was screened, identifying AuNP27 and AuNP35 as potent dendritic cell activators that improve antigen presentation and T cell responses in preclinical cancer models (195). AI also enables personalized vaccine formulations by integrating patient-specific genomic, proteomic, and immunological data (196). By analyzing individual immune profiles, AI can predict optimal LNP compositions and adjuvant combinations, tailoring vaccines to patients with varying immune or tumor microenvironment characteristics. For instance, patients with high inflammation may require lower-reactogenicity formulations, whereas those with immunosuppressive tumors might benefit from stronger adjuvants. This approach allows real-time design of personalized cancer vaccines optimized for maximal immune activation.

## 7 Discussion

Therapeutic mRNA cancer vaccines represent a paradigm shift in precision oncology, yet their clinical success remains constrained by challenges unique to tumor biology, including genomic instability, intratumoral heterogeneity, immune evasion, and inefficient antigen presentation (10). Unlike prophylactic vaccines, which rely on conserved pathogen-derived antigens, cancer vaccines must contend with patient-specific neoantigen landscapes shaped by clonal evolution and immune selection (16). Consequently, the integration of bioinformatics and AI into mRNA vaccine development is not merely an optimization strategy but a necessity for navigating the complexity of tumor-specific immune targeting. AI-enabled neoantigen discovery pipelines have substantially improved the prioritization of candidate epitopes by integrating somatic mutation profiling, RNA expression, HLA typing, and antigen processing constraints. Importantly, emerging DL models trained on immunopeptidomics datasets enable the identification of neoepitopes that are naturally processed and presented on MHC class I and II molecules, addressing a major limitation of earlier sequence-based prediction approaches (58). These advances are particularly relevant in solid tumors, where defective antigen presentation and MHC downregulation represent dominant mechanisms of immune escape. Incorporating clonality, allelic imbalance, and tumor purity into AI frameworks further refines neoantigen selection by prioritizing targets with higher likelihood of uniform tumor expression and sustained immune visibility.

Beyond antigen identification, AI-driven optimization of mRNA constructs plays a critical role in shaping antitumor immune responses. In cancer vaccination, mRNA design parameters including codon usage, untranslated region (UTR) architecture, and secondary structure directly influence translation kinetics, antigen abundance, and proteasomal processing efficiency (108). These features collectively determine the quality of cross-presentation by dendritic cells and the balance between CD8^+^ cytotoxic and CD4^+^ helper T-cell responses. Unlike antibody-focused vaccine endpoints, therapeutic cancer vaccination requires sustained yet controlled antigen expression to avoid T-cell exhaustion while maintaining effective immune priming (4). AI-guided sequence optimization enables rational tuning of these parameters, surpassing empirical design strategies and supporting the development of multivalent neoantigen constructs.

Delivery remains a central bottleneck for mRNA cancer vaccines, particularly in solid tumors characterized by dense extracellular matrix deposition, vascular compression, and elevated interstitial fluid pressure. AI-informed design of lipid nanoparticle (LNP) formulations increasingly incorporates tumor-specific transport barriers, leveraging imaging-derived and biophysical features to optimize nanoparticle size, composition, and surface properties. Integrating delivery modeling with immunogenicity prediction represents an important step toward end-to-end optimization of mRNA vaccine performance within the tumor microenvironment (17).

AI-driven neoantigen prediction has shown promising but variable success in cancer immunotherapy, highlighting the need for experimental validation. High-throughput studies report that only 6–32% of top-ranked neoantigens predicted to bind HLA-A*02:01 elicit measurable T-cell responses (197, 198). However, clinical trials have demonstrated improved outcomes: a poly neoantigen vaccine in melanoma induced T-cell responses against over 60% of included peptides, and an mRNA neoantigen vaccine for pancreatic cancer elicited robust responses correlating with delayed tumor relapse (199). Experimental validation, such as co-culturing patient-derived T cells with predicted peptides, confirms the immunogenicity of AI-selected neoantigens (200). Despite this, most established vaccines still rely on well-characterized epitopes, and successful translation of non-canonical AI-identified epitopes remains limited. Overall, integrating computational predictions with experimental feedback is essential to enhance precision and translational impact in vaccine development. Given the large number of candidate epitopes generated by AI tools, experimental immunologists require clear criteria to prioritize which predictions to pursue. A key best practice is to apply stringent scoring thresholds while understanding the trade-offs in sensitivity. Epitope prediction algorithms typically provide metrics such as binding affinity (IC50 in nM) or percentile rank. Common cut-offs, like IC50 < 500 nM for MHC binders or the top 1–2% rank, are often used to define strong candidates. For example, neoantigen studies frequently retain only mutations predicted to bind HLA with <500 nM affinity and expressed in the tumor (RNA reads ≥1) (95). While such thresholds focus resources on high-likelihood epitopes, overly strict cut-offs can reduce recall; one analysis noted that conventional affinity thresholds captured only ~40% of true binders in empirical datasets. To balance sensitivity and specificity, researchers often use a multi-tiered approach, starting with a lenient filter (e.g., IC50 < 500 nM or top 5% rank) and then prioritizing within this pool using additional criteria, which prevents premature exclusion of biologically important peptides, such as certain CD4+ epitopes with moderate MHC-II affinities (201). It is also important to interpret predicted scores cautiously, as small differences around the cut-off may not reflect meaningful differences in immunogenic potential (202).

Consensus and cross-validation among multiple prediction tools further enhance confidence in selected epitopes. Because outputs from different algorithms (e.g., NetMHCpan versus motif-based methods for T cells, or multiple B-cell predictors) often only partially overlap, candidates ranked highly across independent methods are more reliable. At the same time, relying solely on consensus may omit unique epitopes detected by individual tools, so a balanced ensemble approach prioritizing shared predictions while including some top-scoring unique candidates can improve coverage. Incorporating biological context also improves the relevance of predictions. AI models often overlook factors such as antigen abundance, processing pathways, or immune tolerance, so prioritizing epitopes from highly expressed proteins or conserved pathogen regions enhances *in vivo* significance. For tumor neoantigens, ensuring mutation exclusivity to cancer cells reduces tolerance issues. Consulting immunological databases like IEDB can provide historical validation and guide assay design, while integrating prediction confidence metrics, such as confidence intervals or ensemble variance, helps rank epitopes more reliably (203).

The IEDB-AR (Immune Epitope Database Analysis Resource) is an essential platform for designing mRNA vaccines targeting highly variable antigens. It offers a comprehensive suite of tools for T-cell epitope prediction, B-cell epitope prediction, and analysis of known epitope sequences or epitope groups, making it especially valuable for diseases that require broad or multi-layered immune responses (204). Another major addition is LYRA, an automated framework for modeling 3D structures of B-cell and T-cell receptors, enabling the prediction of canonical receptor conformations when needed (205). SYFPEITHI, one of the earliest freely accessible bioinformatics tools developed in the late 1990s, is widely used for predicting peptide–MHC interactions for both MHC class I and II molecules. Its intuitive interface and strong predictive performance make it valuable for identifying MHC-binding peptides and potential epitopes (206). SYFPEITHI screens peptide sets and predicts epitopes based on sequence motifs, restriction elements, and protein or gene-derived peptide patterns, supporting rational vaccine design. However, its accuracy is inherently tied to the completeness and quality of available peptide–MHC interaction data, and gaps in these datasets may reduce prediction reliability. Using machine learning algorithms such as ANNs and support vector machines (SVMs), IEDB-AR predicts peptide–MHC binding affinities for both class I and II molecules, identifies T-cell and B-cell epitopes, and detects cross-reactive epitopes (204). This allows the platform to identify optimal antigenic regions including pHLA-target antigens capable of eliciting robust CD4^+^ and CD8^+^ T-cell responses alongside antibody responses. However, the reliability of these predictions depends heavily on the breadth and quality of peptide–MHC interaction data. Gaps or inconsistencies within the underlying datasets can affect prediction accuracy and contribute to variability in performance (207).

Moreover, ML models swiftly engage in vaccine pre-clinical and clinical trials. During the preclinical phase, several models are developed to identify peptide-major histocompatibility complex class (MHC) pairing relationships, accurately predict whether an antigenic peptides can be recognized, presented to CD4+ T cells and subsequently trigger a genuine immune response (208–210). Effective integration of AI predictions into experimental workflows benefits from collaboration between computational and laboratory teams. AI analyses need automating data parsing into user-friendly formats (e.g., annotated excel sheets), and maintaining code reproducibility via version control. Providing experimentalists with basic training in command-line tools or FASTA file handling facilitates workflow integration. Regular joint meetings allow teams to review and select predictions collaboratively, ensuring transparency and informed decision-making. To manage the overwhelming volume of AI-generated predictions, phased validation strategies initially testing a manageable subset of top-ranked epitopes before expanding are effective. Early planning for peptide synthesis and quality control is essential, as these processes can be time-consuming and costly. High-throughput approaches, such as using peptide pools in ELISpot assays, accelerate validation but require careful informatics support for deconvolution. Automated systems, including enzyme-linked immunosorbent assay (ELISA) plate readers and barcode labeling, further improve efficiency, supporting a smooth transition from computational predictions to experimental validation (81). Clear communication between computational and experimental teams is critical. Experimentalists should outline practical constraints, such as the number of peptides that can be tested per quarter, enabling computational colleagues to adjust prediction criteria appropriately. Conversely, computational scientists should clarify prediction scores and uncertainties, providing realistic expectations of success. By embedding AI as an iterative, collaborative element within experimental pipelines where wet-lab results continually refine computational models, researchers shift from trial-and-error approaches to efficient, data-driven workflows. Recent studies highlight that such integration accelerates epitope discovery for next-generation vaccines (82, 99). Ultimately, AI serves as a valuable guide rather than a replacement for laboratory work, enhancing precision and efficiency in antigen selection through thoughtful integration and rigorous validation. Integrating AI epitope prediction into vaccine labs requires coordinated collaboration between experimentalists and computational experts, ensuring access to appropriate resources; from standard computers for sequence-based tools to GPUs or cloud platforms for deep-learning models. Proper data formatting and initial testing with known antigens are crucial for accurate predictions, while web-based interfaces can simplify usage but may have privacy or submission limitations.

## 8 Current challenges

Despite significant progress in AI-driven epitope prediction, several challenges remain that researchers are actively addressing. One major limitation is data scarcity and bias: deep learning models depend heavily on the quantity and quality of training datasets, yet epitope data are often limited and skewed toward specific pathogens or common HLA alleles, while rare alleles and negative examples (true non-epitopes) are underrepresented, leading to potential overprediction of epitopes. Strategies such as data augmentation, semi-supervised learning, and transfer learning from general protein datasets have been employed to mitigate these biases (211–213). Another key challenge is generalizability to novel pathogens or rapidly mutating variants. Models trained on well-characterized antigens may perform poorly on distantly related species or emerging viral variants, as observed in cross-validation studies. Continual learning and updating models with newly generated experimental data, as well as incorporating diverse antigen types, are approaches being explored to enhance model robustness, exemplified by frameworks like VenusVaccine (214). Integration of immune context represents an additional hurdle. Most current predictors assess epitopes in isolation, whereas immunogenicity depends on factors such as flanking sequences, pathogen expression levels, host HLA and TCR repertoires, and B-cell lineage availability. Incorporating antigen processing steps (proteasomal cleavage, TAP transport and structural flexibility, particularly of TCR loops, into prediction models is critical for improving accuracy and generalizability (201, 215). Evaluation and validation are further complicated by inconsistent benchmarking: studies employ varying metrics as AUC, AUPR, F1 and datasets, making cross-comparison difficult. Comprehensive benchmarks indicate that many models overfit their training data, limiting predictive performance in real-world scenarios (215). Blind, prospective validation, such as predicting epitopes prior to experimental confirmation, remains essential, as top-ranked epitopes may bind *in vitro* but fail to elicit strong *in vivo* immune responses (216). Structural prediction of short linear peptides, particularly in pMHC contexts, is another major bottleneck. Models like AlphaFold often generate conformations that deviate from experimentally observed structures when peptide sequences differ from training data, underscoring the need for diverse structural datasets to improve generalizability (217, 218). Despite these challenges, AI has significantly advanced epitope prediction by leveraging deep sequence understanding with transformers and structural modeling via graph networks, improving the identification of vaccine targets. Hybrid models and novel training strategies are pushing the limits of prediction accuracy. By addressing data bias, generalizability, and immune-context integration, future AI models could become increasingly reliable, potentially enabling the *in-silico* design of entire vaccine formulations. Such developments promise to accelerate the transition from pathogen discovery to vaccine deployment, highlighting the transformative potential of combining immunology, structural biology, and AI in modern vaccine development (23, 214).

## 9 Emerging trends and novel insights

Beyond core advances, several emerging aspects of AI-driven epitope prediction are shaping the next generation of vaccine design tools. One important development is neoepitope and immunogenicity prediction. Traditional T-cell epitope tools have largely relied on peptide–MHC binding as a proxy for immune response, yet not all binders are truly immunogenic. Modern AI models address this gap by predicting whether a peptide–MHC complex will elicit T-cell responses, while recognizing that high immunogenicity does not guarantee protective or neutralizing effects and may carry risks of auto-reactivity or poly-reactivity. For example, DeepHLApan employs a two-module network to predict both HLA–peptide binding and T-cell activation, improving precision in identifying true neoantigens in cancer immunotherapy (72). Transfer learning is also used on immunopeptidome data to rank peptides based on T-cell recognition likelihood, incorporating factors such as TCR repertoire and antigen processing efficiency. These approaches shift the focus from binding affinity alone to predicting functional immune outcomes. Generative epitope design is another emerging area. Deep generative models, including GANs and variational autoencoders, can produce synthetic peptides resembling known immunogenic epitopes. For instance, DeepImmuno-GAN generated peptides with physicochemical properties and predicted immunogenicity comparable to natural epitopes, expanding the candidate space and suggesting modifications to improve potency or cross-reactivity (81). Complementing this, interpretability methods such as attention weights, saliency maps, and SHapley Additive exPlanations (SHAP) values have been increasingly applied to deep learning models. These approaches highlight key residues, motifs, or structural regions driving predictions, aligning computational outputs with biological understanding. Graph-based models like GraphEPN visualize residue-level scores on 3D antigen structures, revealing immunogenic hotspots to guide experimental validation, while transformer-based language models (e.g., mBLM) identify critical antibody-binding motifs within antigens (219, 220). For T-cell epitopes, attention and attribution methods help pinpoint essential residues or TCR features, ensuring that predictions reflect biologically meaningful interactions rather than artifacts (221, 222). AI is also enabling multi-epitope vaccine optimization, a combinatorial problem where algorithms evaluate millions of epitope sets to balance immunogenicity and population coverage. Deep learning predictors integrated with optimization frameworks, such as genetic algorithms or integer linear programming, can propose theoretical multi-epitope vaccine designs that maximize predicted protection across diverse HLA types and viral strains (106, 223). While largely *in silico*, these approaches illustrate the potential of AI to guide rational vaccine formulation beyond single-epitope selection. Comparative evaluation of AI epitope prediction tools is essential for practical adoption. Key attributes include accessibility (web-based versus standalone software), input requirements (sequence or 3D structural data), and interpretability. Peer-reviewed tools provide quantitative performance metrics such as accuracy, AUC, precision, recall, and MCC, supporting evidence-based selection (215). Structure-informed predictors significantly improve conformational B-cell epitope identification, while sequence-based models, including transformers and recurrent neural networks, efficiently scan pathogen genomes for candidate peptide vaccines and predict T-cell epitopes (224). Output formats that provide residue-level scores mapped onto sequences or 3D structures enhance interpretability, allowing experimentalists to directly link computational predictions to laboratory validation. Accordingly, structure-based tools are preferred for antibody epitope mapping on complex antigens, whereas high-throughput sequence-based predictors are more suitable for peptide vaccine design. By integrating performance metrics, input requirements, and interpretability, researchers can select the most appropriate AI tool for their experimental goals, bridging computational predictions with actionable vaccine development workflows (225, 226).

## 10 Conclusion

mRNA-based cancer vaccines have emerged as a transformative modality in precision oncology; however, their therapeutic success is fundamentally constrained by tumor-specific biological complexities that are absent in prophylactic vaccination. Genomic heterogeneity, clonal evolution, immune escape, and inefficient antigen presentation continue to limit durable clinical responses, underscoring the need for rational, data-driven vaccine design strategies. In this context, the convergence of bioinformatics, AI, and tumor immunology represents a critical advance rather than technological convenience.AI-enabled frameworks now allow systematic integration of tumor–normal sequencing, neoantigen expression, antigen processing, and patient-specific HLA diversity to prioritize clinically actionable targets with improved immunogenic potential. Importantly, the future impact of mRNA cancer vaccines will depend on the successful integration of computational predictions with experimental and clinical validation (227–229). Multimodal AI models that incorporate genomic, transcriptomic, proteomic, spatial, and clinical response data are essential to capture the dynamic interactions between evolving tumors and host immunity. As these models mature, they are expected to enable iterative, patient-specific vaccine refinement and to support combination strategies with immune checkpoint blockade, adoptive cell therapies, and tumor microenvironment–modulating interventions. By explicitly accounting for tumor heterogeneity, antigen processing constraints, and immune resistance mechanisms, AI guided mRNA cancer vaccines hold substantial promise for improving immunogenicity, durability, and clinical efficacy, thereby advancing personalized cancer immunotherapy toward routine clinical implementation.
